# Acute Myelitis, Recurrent Optic Neuritis, and Seizures Over 17 Years

**DOI:** 10.3389/fneur.2020.541146

**Published:** 2020-11-13

**Authors:** Chen Zhao, Aijun Li, Lei Liu, Jiawei Wang, Dongsheng Fan

**Affiliations:** ^1^Department of Neurology, Peking University Third Hospital, Beijing, China; ^2^Department of Ophthalmology, Peking University Third Hospital, Beijing, China; ^3^Beijing Key Laboratory of Restoration of Damaged Ocular Nerve, Peking University Third Hospital, Beijing, China; ^4^Department of Neurology, Beijing Tongren Hospital, Capital Medical University, Beijing, China; ^5^Key Laboratory for Neuroscience, Ministry of Education/National Health Commission, Peking University, Beijing, China

**Keywords:** acute myelitis, optic neuritis, seizures, MOG associated disease, GFAP astrocytopathy, multiple sclerosis

## Abstract

Recent discovery of several autoantibodies, such as aquaporin-4 immunoglobulin G antibodies (AQP4-IgG), myelin oligodendrocyte glycoprotein immunoglobulin G antibodies (MOG-IgG) and glial fibrillary acidic protein immunoglobulin G antibodies (GFAP-IgG), has greatly facilitated differential diagnosis of autoimmune disorders of the central nervous system. Here we report an interesting case with a history as long as 17 years. Only until she was tested positive for MOG-IgG that her diagnosis was revised from multiple sclerosis to MOG-associated disease (MOGAD). Our case illustrates the significance of screening autoantibodies in patients suspected of inflammatory autoimmune neurologic disorders. In addition, this case demonstrates how MOGAD manifests and develops in a patient over a decade.

## Case

**Figure 1:**
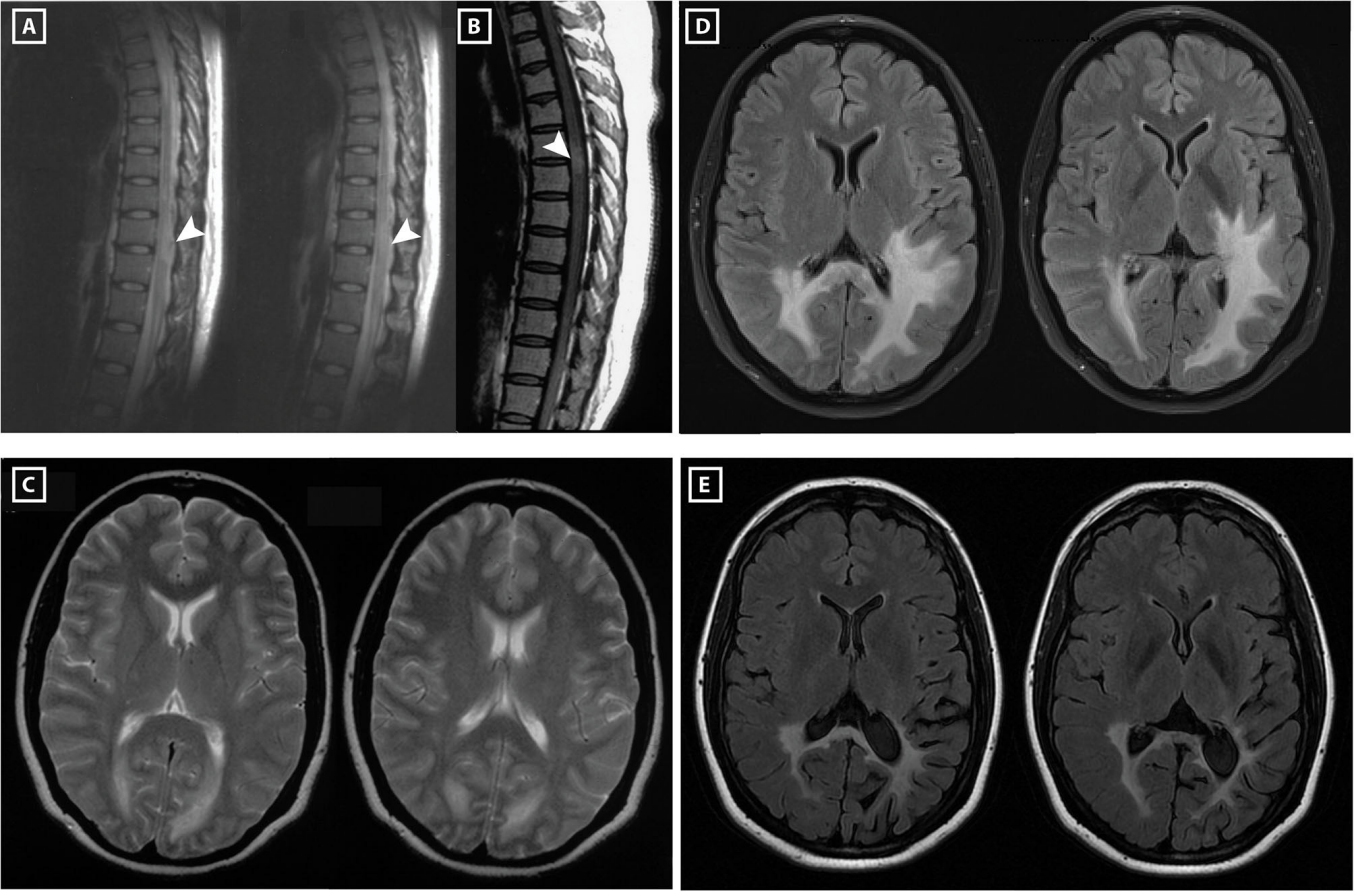
Spinal cord and brain MRI of the patient. In 2003, MRI of the spinal cord showed a hyper-intense lesion at T9–T11 levels **(A)**, and gadolinium-enhanced T1-weighted MRI showed a hyper-intense lesion at T4–T5 levels **(B)**. Her brain MRI revealed periependymal lesions around occipital horns of lateral ventricles **(C)**. In 2009, brain MRI showed T2 hyper-intense lesions around occipital horns of both lateral ventricles extending into left temporal and occipital lobes, right occipital lobe and splenium of the corpus callosum **(D)**. In 2017, brain MRI showed the white matter lesion reduced in size compared to the MRI performed in 2009 **(E)**.

In 2003, a 30-year-old Chinese woman presented with paresthesia and weakness of lower extremities and acute urinary retention after long working hours. Spinal cord MRI showed a T2-weighted hyper-intense lesion at T9–T10 levels and a gadolinium-enhanced T1-weighted lesion at T4–T5 levels (Figures 1A,B). Brain MRI revealed periependymal white matter lesions (Figure 1C). Examination of cerebrospinal fluid (CSF) found no abnormalities in cell counts, glucose level, total protein and IgG index, and zero oligoclonal band (OCB) was detected. Blood investigations of HIV, anti-nuclear antibody, double stranded DNA, extractable nuclear antigens and anti-neutrophil cytoplasmic antibody were all negative. Following treatment with intravenous methylprednisolone pulse (IVMP, 1 g for 3 days and gradually tapered) and intravenous immunoglobulins (IVIG, 20 g for 5 days), the patient recovered completely 2 months later.

**Figure 2:**
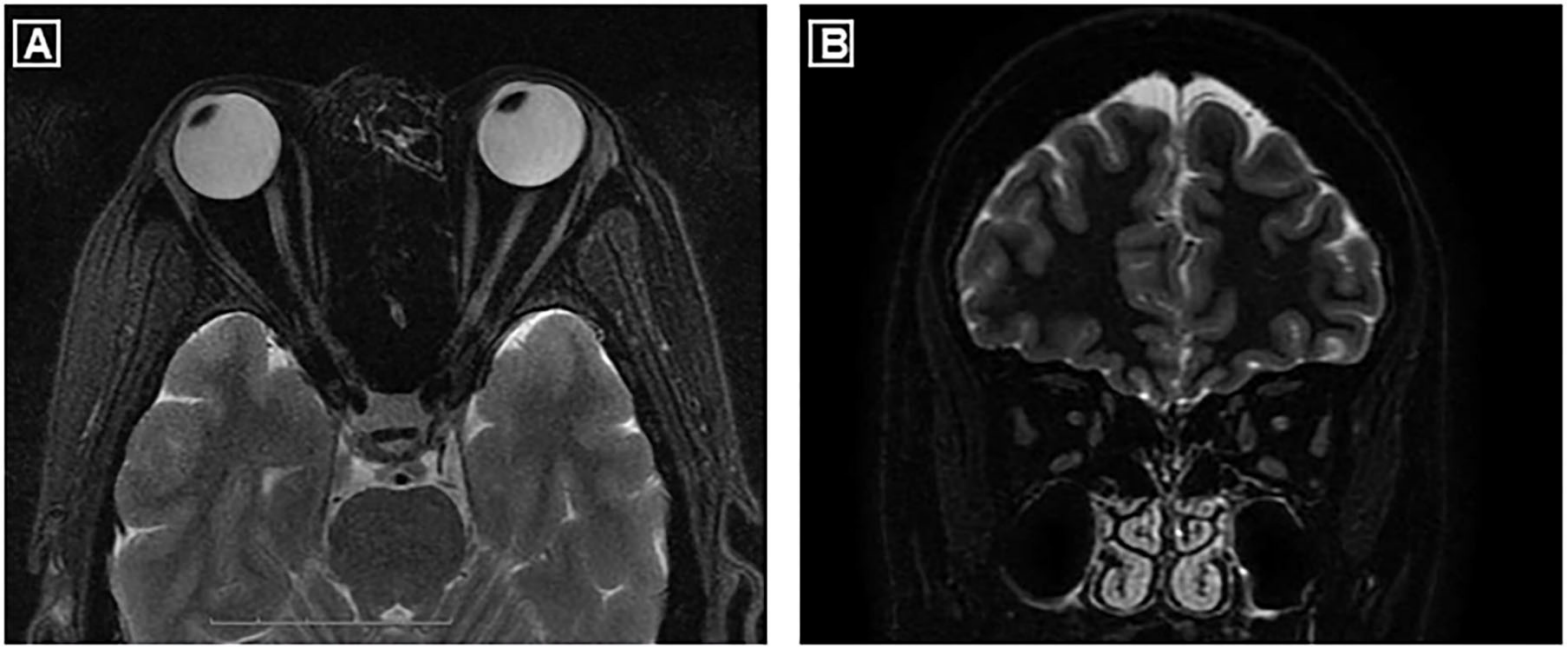
Axial **(A)** and coronal **(B)** MRI scans of optic nerves showed bilateral atrophy.

Over tapering to 5 mg oral prednisone every other day, she presented with sudden vision blurring and pain on movement of her left eye at 5 months after acute myelitis. Visual field test revealed peripheral vision defect in her left eye. Visual evoked potential showed reduced P100 amplitude in both eyes. She was treated with retrobulbar steroid injection and IVIG with a full vision recovery. From 2004 to 2009, she suffered recurrent unilateral/bilateral optic neuritis (ON), with a frequency of 1–2 episodes/year. Notably, a brain MRI in 2009 showed massive T2 hyper-intense white matter lesions (Figure 1D). For each episode, her vision usually had complete recovery after treatment with either retrobulbar steroid injection or IVMP, occasionally in combination with IVIG. However, over these relapses her vision field defect slowly progressed from a blurred point to the right inferior quadrant in both eyes. MRI of optic nerves conducted in 2016, 13 years after the first ON attack, showed bilateral atrophy (Figures 2A,B). Following the treatment with interferon-beta initiated in 2009, frequency of ON reduced to 1 episode/2 years. Her visual field defect did not progress, and her vision remained 1.0 in both eyes.

In 2010 and 2011, she experienced two episodes of generalized seizures, and oral valproic acid was initiated. In 2018, another episode of generalized seizures occurred. Since 2015 she started to suffer left parietal headache which could relieve after rest. In 2017, she suffered sudden numbness of upper and lower extremities, lasting for 6 h before complete recovery. Brain MRI did not reveal new lesions (Figure 1E). Additionally, a teratoma (0.5 × 0.4 cm) was found in her left ovarian which has not been removed by surgery.

**Figure 3:**
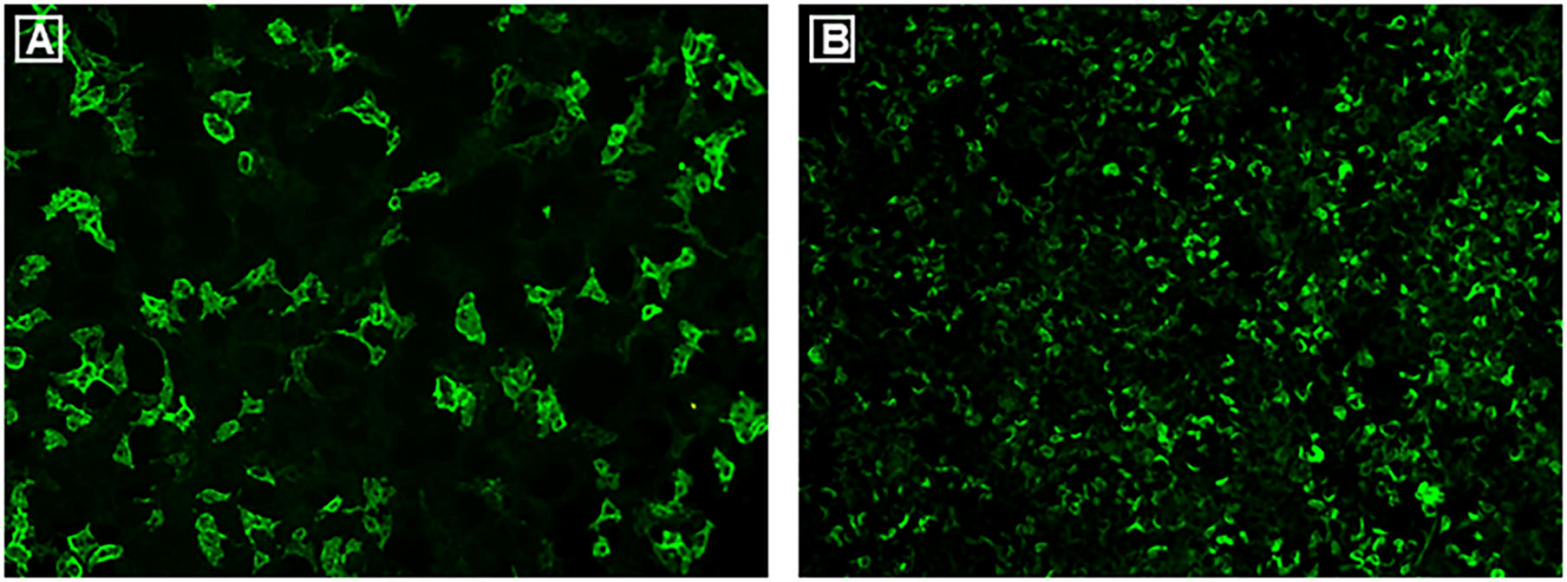
Commercial CBAs were used to examine autoantibodies in serum in 2020. Both MOG-IgG and GFAPα-IgG were positive with titers of 1:320 **(A)** and 1:100 **(B)**, respectively.

Her frequent bilateral ON relapses and extensive white matter lesions in brain prompted re-consideration of the multiple sclerosis (MS) diagnosis. Therefore, investigation of autoantibodies was first performed in 2016. Three independent tests of aquaporin-4 immunoglobulin G antibodies (AQP4-IgG) in serum using cell-based assays (CBA, Euroimmun AG, Germany) were all negative. Although myelin oligodendrocyte glycoprotein immunoglobulin G antibodies (MOG-IgG) were detected in serum, the titer was merely 1:10. In 2017, following discovery of the glial fibrillary acidic protein immunoglobulin G antibodies (GFAP-IgG) (1, 2), examination of this autoantibody in CSF and serum was performed. A tissue-based assay (TBA) using rat cerebellum and hippocampus showed the characteristic GFAP staining pattern of her serum. In 2020, examination of autoantibodies in serum was repeated in order to confirm the diagnosis. The patient was seropositive for MOG-IgG with a titer of 1:320 (Figure 3A), whereas AQP4-IgG remained negative (CBA, Euroimmun AG, Germany). GFAP-IgG was detected with a titer of 1:100 using CBA (Shaanxi MYBiotech Co., Ltd. China, Figure 3B). Given the presence of teratoma, antibodies related to autoimmune encephalitis (NMDAR, LGI1, GABABR, CASPR2, AMPA1, AMPA2, DPPX) were all tested with negative findings. CSF samples were unavailable due to the patient's refusal of a lumbar puncture.

## Discussion

For a prolonged period, this patient was diagnosed with MS, but absence of OCB and lack of the typical Dawson fingers pattern has challenged the diagnosis. Her Asian ethnicity and frequent ON relapses raised the possibility of neuromyelitis optica spectrum disorders (NMOSD) (3). However, repetitive testing of AQP4-IgG was negative in serum, and her spinal cord lesion did not meet the diagnostic criteria for NMOSD without AQP4-IgG (3). The first examination of MOG-IgG revealed a very low titer of 1:10 in serum. Only until the recent test found a titer of 1:320 in serum that her diagnosis was revised to MOG-associated disorder (MOGAD).

MOG was first identified as a target in inflammatory demyelinating response in animal models (4). As the specificity of CBAs for MOG-IgG improving, a proportion of patients with atypical MS phenotypes have been revealed. Clinical manifestations of MOGAD are characterized by acute disseminated encephalomyelitis (ADEM) in children and opticospinal phenotypes in adults, including ON, myelitis and brainstem encephalitis (5). Some patients meet the diagnostic criteria of AQP4-seronegative NMOSD (3). ONs are the most common phenotype in relapsing patients, and seizures have also been reported to be associated with MOGAD (6). Our patient's manifestations are well compatible with MOGAD in adults. Her relatively mild disability after a long-term disease course is also a feature of this disease (5). The extensive white matter lesions found in her brain, although rarely observed in adults, have been reported in some cases (7). Of note, interferon-beta seemed to be beneficial to our patient, as it reduced the frequency of ON from 1 to 2 episodes every year to 1 episode every 2 years. This appears to contradict some studies which found disease-modifying medications of MS being ineffective or even harmful to patients with MOGAD (8). The ethnicity difference between our patient and the reported Caucasian cases could be one possible explanation. However, further investigation with a larger group of patients is required before drawing a firm conclusion.

Interestingly, GFAP-IgG was tested positive in her serum in 2017 and 2020, using TBA and CBA, respectively. GFAP astrocytopathy is a recently defined spectrum disorder of autoimmune neurologic syndromes characterized by necrotizing meningoencephalomyelitis (1). The most common clinical phenotypes include encephalopathy, myelitis, headache, seizures, blurred vision and maliganancy (9), some of which are presented in our case. The seropositivity of GFAP-IgG raises the possibility that both MOG-IgG and GFAP-IgG play a role in pathogenesis in our case. Indeed, co-existence of MOG-IgG and GFAP-IgG has previously been reported in Chinese patients (10, 11). Repeated tests of GFAP-IgG in CSF would be helpful in determining whether the patient has overlapping MOGAD and GFAP astrocytopathy. Unfortunately, this has been hampered by the patient's refusal to repeat a lumbar puncture.

Overall, our case demonstrates how MOGAD manifests on a patient with detailed follow-up as long as 17 years. It illustrates the importance of screening autoantibodies in patients suspected of autoimmune neurologic disorders, especially when they present with atypical phenotypes. It becomes clearer that MOGAD is distinctive from MS and AQP4-IgG-positive NMOSD. Further studies are required to extend our understanding of phenotypes and pathogenesis of these syndromes.

## Data Availability Statement

The datasets generated for this study are available on request to the corresponding author.

## Ethics Statement

Written informed consent was obtained from the individual(s) for the publication of this case report, including any potentially identifiable images or data included in this article.

## Author Contributions

DF conceived this study and provided financial support. DF and CZ designed the study. AL took part in the design of the study and in sample collection. CZ and AL conducted data follow-up and provided Figure 1. LL examined the patient in 2016, performed all the autoantibodies testing since year 2017 and provided Figures 2, 3. CZ, LL, and JW undertook data checking. CZ, LL, and DF were responsible for preparing and revising the manuscript. All authors contributed to the article and approved the submitted version.

## Conflict of Interest

The authors declare that the research was conducted in the absence of any commercial or financial relationships that could be construed as a potential conflict of interest.
